# Exploration of Novel Anti-Oxidant Protein Sestrin in Frailty Syndrome in Elderly

**DOI:** 10.14336/AD.2017.0423

**Published:** 2018-04-01

**Authors:** Nitish Rai, G. Venugopalan, Rashmita Pradhan, Akash Ambastha, Ashish Datt Upadhyay, Sadanand Dwivedi, Aparajit B. Dey, Sharmistha Dey

**Affiliations:** ^1^Department of Biophysics,; ^2^Department of Geriatric Medicine,; ^3^Department of Neurology,; ^4^Department of Biostatistics, All India Institute of Medical Sciences, New Delhi - 110029, India

**Keywords:** Serum, Surface Plasmon Resonance, Protein marker, Immunoblot

## Abstract

Frailty in elderly is very much familiar with a decline in the musculoskeletal system. Muscle degeneration in the lower organism was observed due to loss of anti-oxidant protein Sestrin. The aim of the study is to determine the level of Sestrin1 and Sestrin2 in the serum of frail and non-frail elderly to associate their impact in frailty syndrome. Subjects with age ≥ 65 years were enrolled from Geriatric Medicine OPD of All India Institute of Medical Sciences, New Delhi (N= 92). Among them, 51 subjects were identified as frail and rest 41 were regarded as non-frail according to “deficit accumulation model of Rockwood.” The study was performed by surface plasmon resonance and validated by western blot. Sestrin1 and Sestrin2 were found to be significantly reduced in frail compare to non-frail elderly. Furthermore, even after the adjustment for age, gender and education, the level of Sestrin1 and Sestrin2 remain significantly lower across the groups. The Sestrin1 level was significantly lower in various categories like age, gender, BMI, education, ADL, number of co-morbidity along with other clinico-pathological features. ROC analysis also revealed the distinction of frail and non-frail in respect to serum Sestrin1 and Sestrin2. This study highlighted the new and promising role of serum Sestrin in frail and non-frail elderly. In future, it can be utilized as molecular marker to assess the potential diagnostic value for clinical purpose.

The success of modern medicine in curing acute illnesses and managing chronic conditions has increased life expectancy across the world [1]. The dramatic increase in the numbers of older people, coupled to the continuing presence of chronic diseases, can compromise physical functioning for many, which manifests as frailty. Aging, due to an accumulation of damaged molecules, cells and tissues over a lifetime, often leads to frailty. Functional decline, disability and frailty are the common conditions across geriatric syndromes. Frailty syndrome is complex and characterised by a compromised capacity to resist physical challenges and homeostatic distress [2]. According to Fried, frailty is defined by unintended weight loss, muscles weakness, fatigue, low levels of activity and slow gait capacity. Sarcopenia, the loss of skeletal muscle mass, is a major contributor to frailty syndrome, which is associated with increased falls, dependency and mortality [3,4]. Frailty can also be assessed by Rockwood’s criteria, which encompasses accumulated deficits over age, in association with a high risk of mortality and adverse outcomes [5]. The cumulative deficit model includes 36 variables that provide a frailty index [6]. The Fried phenotype and Rockwood Cumulative scales are widely used in the clinical assessment of frailty.

Despite its substantial impact on the health and functioning of the elderly population, the pathophysiology of frailty and its surrogate marker, sarcopenia has yet to be determined. The development of biomarkers for these conditions, despite being extremely challenging, is of great importance [7]. A biomarker would provide a new way of defining disability outcomes in epidemiological studies of the elderly that is based on underlying pathophysiological processes. Early identification of frailty would help to reduce the morbidity and mortality in older adults, as well as adding to quality of life. Identifying the onset of frailty, before any clinical manifestations, may provide a window of opportunity for intervention that decreases the emergence of frailty.

A blood-based protein marker may be preferred, given that it is non-invasive, easily accessible and without major discomfort for the frail elderly. Different body proteins may ultimately accumulate in various biological fluids, thereby aiding the identification of early disease stages. Although a few biomarkers have been proposed in various cohorts of frail people, some are not directly pathophysiology-related or have failed to prevent frailty when supplemented [8]. The search for biomarkers in the field of frailty is an area of active research, with some importance, given that frailty in older adults can be prevented or reversed, if detected early [9].

Physical exercise seems to be most effective approach for delay, and sometimes prevention, of frailty in the elderly [10-14]. Increased reactive oxygen species (ROS) enhances levels of oxidative damage in cell plasma membranes, DNA and other molecules, with ROS being increased by exercise [15]. However, physical exercise also increases endogenous antioxidants, including the peroxiredoxins (PRXs) system, which counter the effects of ROS. Sestrin (Sesn) is a stress inducible protein that controls the PRX system, and which may be modulated by physical exercise [16,17]. In addition, Sesn is clearly associated with aging related processes, including AMP-activated protein kinase (AMPK), the mechanistic target of rapamycin (mTOR), p53, Fork-head box proteinO (FOXO) and PRXs signalling pathways, which are strongly related to aging processes. The optimized functioning of these pathways is therefore important to the prevention of age related pathophysiologies that underpin sarcopenia and frailty. Exercise also modulates these pathways, being the most highly recommended strategy to prevent sarcopenia and frailty, increase longevity, and promote sound health in the elderly. Loss of Sesn can cause several chronic pathologies, such as mitochondrial dysfunction, muscle degeneration, fat accumulation and cardiac arrhythmia in drosophila [18]. To date, the relationship of Sesn with frailty has not been explored. This study assesses the impact and potential utility of Sestrin1 and Sestrin2 in the diagnosis of frailty by determination of their serum levels in frail and non-frail elderly.

## MATERILAS AND METHODS

### Participants

The study included 92 participants above the age of 65 years, who were recruited from Geriatric Medicine OPD of the All India Institute of Medical Sciences, New Delhi. The study was conducted as per protocol and was approved by the Institute Ethics Committee (IESC/T-35/03.01.2014). Informed written consent was obtained from all study participants.

Participants were assessed for baseline physical and biochemical characteristics. All participants underwent a comprehensive geriatric assessment (CGA) of frailty. A diagnosis of frailty was made according to the “deficit accumulation model of Rockwood” and a Frailty Index was derived by administration of a 36-item questionnaire [6]. Thirty-six variables indicated health status at baseline, which included medical conditions, health attitudes, symptoms and functional impairments. The Frailty Index does not necessarily include exactly the same deficit variables or the same number of variables. Each variable in the Frailty Index is biologically well founded, accumulates with age, and does not saturate too early in life. One point was scored for each positive response or deficits and 0 represent the absence of any problem. The “deficits” included in the index are common health issues in old age, including across race and socio-economic factors, which have been validated in a wide range of populations. The Frailty Index was calculated as the proportion of the sum of a number of deficits (n) out of total number variables (n/36). Participants with a score of ≥ 0.25 (9 or more positive response) were categorized as frail (n=51), with the remainder of the participants categorized as non-frail (n=41).

Seriously ill participants, including as arising from dementia, delirium, end stage kidney disease and terminal illnesses, were excluded from the study.

### Collection of Blood sample

Having obtained consent and following a complete clinical assessment, 2 ml of venous blood was collected aseptically and allowed to clot in a vertical position for 1 h. After this incubation, blood was centrifuged at 800g for 10 mins and serum was separated. The serum samples were stored at -80 ºC in multiple small aliquots to avoid unnecessary thaw and refreeze. For analysis, the serum specimen was gradually thawed once and analysed immediately.

### Estimation of serum Sesn1 and Sesn2 level

#### by Surface Plasmon Resonance (SPR)

Biacore 3000 system (Wipro GE Healthcare, UK), was used to quantify Sesn level in the serum of study participants. It provides a unique platform for analysis of specific bimolecular interaction in a real-time, label free manner. The CM5 sensor chip (Wipro GE Healthcare, UK), was docked onto the system before the mouse anti-human Sesn1 IgG (Santa Cruz Biotechnology, USA) and rabbit anti-human Sesn2 IgG (Santa Cruz Biotechnology, USA), were immobilized using the amine coupling reaction kit (Wipro GE Healthcare, UK) in separate flow cells of the chip [19].

The different concentrations of recombinant Sesn1 and Sesn2 proteins were passed over their respective flow cell with an immobilized antibody that interacted specifically with the protein and generated an SPR signal (measured as response unit, RU). The standard curve was generated by plotting the response unit, RU, against known concentrations of recombinant Sesn1 and Sesn2 proteins. The serum samples were diluted (1:70) in HBS EP buffer before running on the sensor chip and generated RU was noted. The serum protein concentrations of Sesn1 and Sesn2 in all the samples were determined from their respective standard curve. All the interaction experiments were performed at 25 ºC with HBS-EP (Wipro GE Healthcare, UK) as running buffer.

#### By Immunoblot analysis

A total of ten serum samples, five from the frail and non-frail groups, were randomly chosen for immunoblot analysis. The samples were initially processed by removing the interfering serum albumin protein using the Albumin out kit (G Biosciences, USA) followed by protein quantification using bicinchoninic acid assay (BCA). The processed serum proteins were finally separated using SDS-PAGE and transferred to polyvinylidenedifluoride (PVDF) membranes. The membranes containing transferred proteins were blocked using 5% non-fat milk (Amresco, USA). Then, the membranes were incubated with primary mouse anti-human Sesn1 IgG (1:200) and primary rabbit anti-human Sesn2 IgG antibody (1:400), followed by incubation with secondary Horse Radish Peroxidase (HRP) conjugated with goat anti-mouse IgG and goat anti-rabbit IgG (1:4000), respectively. The membranes were developed and visualized using an enhanced chemiluminescent system (Thermo Scientific, USA) and band density was quantified using the myImageAnalysis Software (Life Technologies).

**Table 1:** Demographic data of frail and nonfrail subjects: age, BMI, gait speed and grip strength illustrated as mean ± S.D.

	Frail	Nonfrail	p-value
**N**	51	41	
**Age**	76.55 ± 6.82	76.12 ± 5.57	0.747
**Female** n (%)	30 (58.8)	8 (19.5)	0.000
**Male** n (%)	21 (41.2)	33 (80.5)	0.000
**BMI** (Kg/m^2^)	22.57 ± 5.09	23.26 ± 4.21	0.492
**Gait speed** (m/s)	0.44 ± 0.16	0.72 ± 0.14	0.000
**Grip strength** (Kg)	12.19 ± 8.83	32.24 ± 13.58	0.000
**OA knee** n (%)	23 (45.1)	10 (24.4)	0.04
**Joint pain** n (%)	25 (49.0)	13 (31.7)	0.094
**Fall** n (%)	17 (33.3)	5 (12.2)	0.018
**Sleep disorder** n (%)	13 (25.5)	2 (4.9)	0.008
**ADL**	17.72 ± 3.20	19.73 ± 0.59	0.000
**Malnutrition** n (%)	45 (88.2)	12 (29.3)	0.000

ADL = activities of daily living; BMI = body mass index; OA knee = osteoarthritis knee; SD = standard deviation.

**Figure 1.**
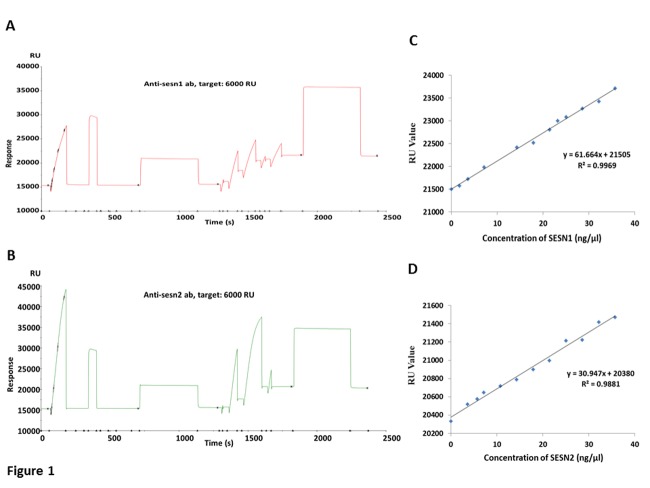
Surface Plasmon Resonance. Immobilization of (A) anti-human Sesn1 IgG and (B) anti-human Sesn2 IgG on the CM5 sensor chip. The linear standard curve, obtained by plotting different response unit (RU) against their respective concentration, showing the binding of increasing concentration of purified (C) Sesn1 and (D) Sesn2 with their respective antibody, resulting in a corresponding increase in RU values.

### Statistical Analysis

Statistical analysis was performed using SPSS Statistics version 17.0 and Graph Pad version 5.0. The statistical analysis was done using Pearson chi-square test for categorical variables and Student’s t-test for comparison of continuous variables. The groups with unknown distribution of data were compared by non paramatric test. The groups, adjusted for age, gender and education, were compared using analysis of covariance. A p-value below 0.05 was considered statistically significant.

**Table 2:** Unadjusted and Adjusted (for age, gender and education) serum protein levels (mean ± S.E.) (95% confidence interval) (ng/µl) of frail and nonfrail subjects.

Protein	Unadjusted/Adjusted	Frail	Nonfrail	p-value
**Sesn1**	Unadjusted	14.58 ± 0.34 (13.9-15.3)	17.61 ± 0.55 (16.5-18.7)	0.000
	Adjusted	14.69 ± 0.43 (13.8-15.5)	17.43 ± 0.48 (16.5-18.4)	0.000
**Sesn2**	Unadjusted	12.74 ± 0.30 (12.1-13.3)	14.14 ± 0.41 (13.3-14.9)	0.003
	Adjusted	12.8 ± 0.36 (12.1-13.5)	14.06 ± 0.40 (13.2-14.9)	0.030

SE = standard error; sesn1 = sestrin1; sesn2 = sestrin2

## RESULTS

### Baseline Data

The demographic data is shown (Table 1), following the complete clinical assessment and diagnosis of the study participants (Table 1). Among the 92 participants, 51 formed the frailty group and 41 the non-frail group. The mean age of the frail group (76.55 ± 6.82 yr) was not statistically different to that of the non-frail (76.12 ± 5.57 yr). Most of the frail subjects were women (58.8%). The gait speed, grip strength, and activities of daily living (ADL) were significantly lower (p<0.0001) in the frail than non-frail group. The frail group were significantly more affected with knee osteoarthritis (OA), number of falls, sleep disorder and malnutrition.

**Figure 2.**
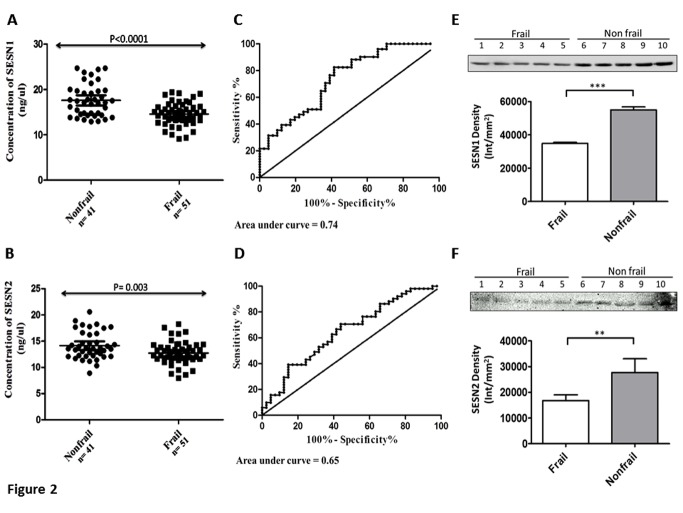
Serum Sestrin level. Scatter graph showing the levels (ng/µl) of serum (A) Sesn1 and (B) Sesn2 in frail (n=51) and nonfrail (n=41) subjects. The serum concentration, estimated by SPR technology, showed a significant decline of Sesn1 (p<0.0001) and Sesn2 (p = 0.003) in frail compare to nonfrail. ROC analysis showing the area under curve for (C) Sesn1 and (D) Sesn2 to differentiate between frail and nonfrail. The area under curve for Sesn1 = 0.74 and Sesn2 = 0.65. The western blot followed by density analysis was performed to confirm the differential expression of (E) Sesn1 and (F) Sesn2 in the serum of frail and nonfrail subjects. Lane 1-5: frail; Lane 6-10: nonfrail. For E and F, values are expressed as Intensity/mm^2^ for frail and nonfrail

### Estimated serum Sestrin level

The immobilization of Sesn1 and Sesn2 antibody was performed effectively, indicated by the sensorgram, with the obtained response being 6075 RU (Fig. 1A) and 4949RU (Fig. 1B), respectively. The standard curves, obtained by plotting different response units against their respective concentration, were found to be linear for both proteins (Fig. 1C and 1D). Low Sesn1 and Sesn2 levels were significantly associated with frailty as shown in Table 2. The serum Sesn1 concentration was significantly low (p<0.0001) in the frail group (14.58 ± 0.34 ng/µl, 95% CI: 13.9-15.3 ng/µl), as compared to nonfrail group (17.61 ± 0.55 ng/µl, 95% CI: 16.5-18.7 ng/µl) (Fig. 2A). Similarly, serum Sesn2 was significantly low (p = 0.003) in the frail group (12.74 ± 0.30 ng/µl, 95% CI: 12.1-13.3 ng/µl) as compared to nonfrail group (14.14 ± 0.41 ng /µl, 95% CI: 13.3-14.9 ng/µl) (Fig. 2B). Furthermore, following adjustment for age, gender and education, the level of Sesn1 and Sesn2 remain significantly lower in the frail group (Table 2). The Sesn1 level was significantly lower in various recorded categories, including age, gender, BMI, education, ADL, number of comorbidities and other clinico-pathological features, in contrast to the mostly non-significant changes in Sesn2 (Table 3). The receiver operating characteristic (ROC) curve was constructed from SPR data, in order to analyse the potential of Sesn1 and Sesn2 as a marker for diagnosis of frailty. The area under curve for predicting frailty was 0.74 for Sesn1 (Fig. 2C) and 0.65 for Sesn2 (Fig. 2D). The cutoff value of Sesn1 calculated for the detection of frailty was ≥15.40 ng/µl, with a sensitivity of 65% and specificity of 66%, while the threshold value of Sesn2 was calculated out to be ≥13.17 ng/µl with a sensitivity of 63% and specificity of 61%.

**Table 3:** The concentration of serum Sesn1 and Sesn2 (ng/µl) as mean ± SD with different clinico-pathological features.

		Sesn1			Sesn2	

	Frail	Nonfrail	p-value	Frail	Nonfrail	p-value
Age						
65-74	15.50 ± 2.36	18.19 ± 3.85	0.010	13.04 ± 2.0	14.45 ± 2.51	0.040
75-79	14.75 ± 2.87	18.21 ± 3.56	0.005	12.78 ± 2.57	14.52 ± 3.14	0.060
≥80	13.67 ± 2.05	16.46 ± 3.15	0.001	12.47 ± 2.03	13.49 ± 2.13	0.080
Gender						
Male	14.71 ± 2.10	17.71 ± 3.47	0.000	12.96 ± 1.45	14.21 ± 2.65	0.026
Female	14.48 ± 2.75	17.22 ± 4.05	0.015	12.59 ± 2.55	13.86 ± 2.52	0.110
BMI						
≤23	14.92 ± 2.46	18.23 ± 3.75	0.002	12.31 ± 1.85	14.16 ± 2.77	0.011
>23	14.39 ± 2.51	16.96 ± 3.27	0.0011	12.98 ± 2.29	14.13 ± 2.46	0.040
Education (Years)						
0	14.81 ± 2.69	17.59 ± 3.38	0.016	13.02 ± 2.14	14.04 ± 2.26	0.228
1-8	15.19 ± 1.99	16.23 ± 2.49	0.257	12.76 ± 2.18	12.81 ± 1.98	0.953
9- 12	12.84 ± 2.93	19.30 ± 4.29	0.028	11.06 ± 2.27	15.86 ± 3.03	0.016
≥13	13.29 ± 1.66	17.76 ± 3.83	0.009	12.61 ± 2.08	14.29 ± 2.60	0.357
ADL						
Impaired	14.56 ± 2.73	17.97 ± 4.02	0.003	12.71 ± 2.35	14.45 ± 3.72	0.048
Normal	14.63 ± 1.75	17.52 ± 3.48	0.003	12.84 ± 1.59	14.07 ± 2.31	0.038
No. of comorbidity					
1	14.49 ± 2.36	17.13 ± 3.36	0.001	12.78 ± 2.09	13.81 ± 2.37	0.042
>1	14.72 ± 2.73	18.44 ± 3.81	0.001	12.69 ± 2.30	14.72 ± 2.93	0.015
Grip strength					
Weak	14.62 ± 2.57	18.11 ± 3.87	0.000	12.75 ± 2.21	14.83 ± 2.89	0.006
Normal	14.13 ± 1.13	17.38 ± 3.43	0.056	12.67 ± 1.64	13.83 ± 3.43	0.266
Gait speed						
Slow	14.63 ± 2.55	15.73 ± 2.44	0.447	12.71 ± 2.23	12.39 ± 1.42	0.682
Normal	14.21 ± 2.01	17.93 ± 3.63	0.007	13.03 ± 1.54	14.45 ± 2.64	0.209
Joint pain						
Yes	14.34 ± 2.69	17.21 ± 3.32	0.003	12.53 ± 2.51	13.84 ± 2.24	0.062
No	14.81 ± 2.29	17.80 ± 3.68	0.000	12.95 ± 1.75	14.29 ± 2.77	0.020
OA knee						
Yes	15.19 ± 2.63	17.59 ± 3.83	0.023	13.34 ± 2.59	14.52 ± 3.04	0.013
No	14.07 ± 2.27	17.62 ± 3.51	0.000	12.25 ± 1.59	14.02 ± 2.47	0.001
Fall						
Yes	14.05 ± 2.35	18.76 ± 4.58	0.031	12.47 ± 2.19	14.74 ± 4.52	0.170
No	14.84 ± 2.53	17.45 ± 3.42	0.000	12.89 ± 2.15	14.06 ± 2.30	0.015

ADL = activities of daily living; BMI = body mass index; OA knee = osteoarthritis knee; sesn1 = sestrin1; sesn2 = sestrin2.

Immunoblotting was performed to further confirm the differential expression of Sesn1 and Sesn2 in the frail and nonfrail groups. Western blot, consistent with SPR data, showed reduced levels of Sesn1 (Fig. 2E) and Sesn2 (Fig. 2F) in the sera of the frail compare to nonfrail group.

## DISCUSSION

Metabolic dysfunction and muscle disorder occur frequently in the elderly. Therefore, the maintenance of muscle strength and healthy mobility in the elderly is necessary for optimal functioning. The onset of frailty brings changes in various organs that contribute to the differential expression and function of an array of proteins. Physical exercise positively regulates PRX system, which is a major scavenger for intacellular hydrogen peroxide thereby exerting a protective effect against oxidative stress-induced damage [20]. Physical exercise has shown to upregulate the PRX isoforms in skeletal and heart muscle cells [21]. Inactivated or mutated Sesn in preclinical studies of *C. elegans* and *Drosophila* shows muscle degeneration [18, 22]. Physical exercise can upregulate Sesn, which inhibits mTOR and prevents age related frailty symptoms [17]. Sesn are regulated by p53, with serum p53 increased after vigorous exercise [23]. Gene silencing of Sesn1 and Sesn2 results in ROS accumulation, highlighting the importance of Sesn1 and Sesn2 in endogenous antioxidant regulation [16]. The exercise-related benefits could be attributed to the antioxidant and AMPK-modulating functions of Sesn.

This preliminary study, for the first time, quantified serum Sesn1 and Sesn2 levels in frail and nonfrail groups by SPR technology, in order to investigate whether Sesn may act as serum protein marker for the clinical diagnosis of frailty. SPR is an advanced technology that facilitates protein detection and quantification in a label-free, in real-time manner at picomolar concentrations. The value of serum analysis for identification of potential protein biomarkers lies in its simplicity, cost effectiveness and safety, as well as its viability for large scale screening. Thousands of translationally modified proteins from different tissues escape into the circulation through leakage or secretion [24,25]. The normal human physiological state is characterized by a different level of such serum proteins, as compared to a pathophysiological state [26,27]. A number of biomarkers have been proposed in various cohorts of frail people, including insulin-like growth factor-1, vitamin D, vitamin B12, albumin, C-reactive protein, haemoglobin, albumin, several hormones, cholesterol, haemoglobin, monocytes, transaminase [8]. Physical exercise and proper nutrition can successfully modulate frailty [28].

Lower Sesn1 and Sesn2 levels were observed in the frail as compared to nonfrail group, including when adjusted for age, gender and education. Sesn are abundantly expressed in skeletal muscles and important in maintaining muscle homeostasis. In drosophila Sesn (dSesn)-null flies are affected with age-associated skeletal muscle degeneration, in association with accumulations of degenerated sarcomeres and dysfunctional mitochondria [18]. Similarly, Sesn-1 mutant worms show raised ROS levels and impaired body muscle function [22]. Such data strongly suggest that Sesn also play a protective role against muscle degeneration in mammals, indicating an important role in normal aging and muscle homeostasis. Our findings are in the agreement with previous studies suggesting that a decline in Sesn may result in muscle weakness, abnormal gait, and frailty.

Consistent with the SPR data, western blot confirmed the decline of Sesn1 and Sesn2 in the frail when compared to nonfrail group. Serum Sesn1 level is lower in the frail group sample aged above 80 years as compared to other age group of the study. Similarly, frail individuals with higher BMI (BMI > 23) had lower Sesn1 level than normal weight individuals. The level of Sesn1 is lower in the frail with impaired activities of daily living (ADL) and in those having a history of falls. These observations indicate that declined Sesn1 levels cause more loss of muscle mass, thereby rendering elderly persons more inactive and frail. The ROC curve indicates that the Sesn level can differentiate the frail from the nonfrail group, with Sesn1 having a higher specificity and sensitivity than Sesn2 for the detection of frailty.

In conclusion, the present study reports that low serum Sesn1 and Sesn2 concentrations are associated with frailty, indicating Sesn as a potential protein marker for the detection of frailty. It may give the important insights about the role of Sesn in the progression of frailty as well as a relevant target for therapeutic interventions.
